# The Methylation Pattern for Knee and Hip Osteoarthritis

**DOI:** 10.3389/fcell.2020.602024

**Published:** 2020-11-06

**Authors:** Zhen Wu, Lu Shou, Jian Wang, Tao Huang, Xinwei Xu

**Affiliations:** ^1^Departmemt of Orthopaedics, Tongde Hospital of Zhejiang Province, Hangzhou, China; ^2^Departmemt of Pneumology, Tongde Hospital of Zhejiang Province, Hangzhou, China; ^3^Shanghai Institute of Nutrition and Health, Chinese Academy of Sciences, Shanghai, China

**Keywords:** osteoarthritis, methylation, Support Vector Machine, minimal Redundancy Maximal Relevance, Incremental Feature Selection

## Abstract

Osteoarthritis is one of the most prevalent chronic joint diseases for middle-aged and elderly people. But in recent years, the number of young people suffering from the disease increases quickly. It is known that osteoarthritis is a common degenerative disease caused by the combination and interaction of many factors such as natural and environmental factors. DNA methylations reflect the effects of environmental factors. Several researches on DNA methylation at specific genes in OA cartilage indicated the great potential roles of DNA methylation in OA. To systematically investigate the methylation pattern in knee and hip osteoarthritis, we analyzed the methylation profiles in cartilage of 16 OA hip samples, 19 control hip samples and 62 OA knee samples. 12 discriminative methylation sites were identified using advanced minimal Redundancy Maximal Relevance (mRMR) and Incremental Feature Selection (IFS) methods. The SVM classifier of these 12 methylation sites from genes like MEIS1, GABRG3, RXRA, and EN1, can perfectly classify the OA hip samples, control hip samples and OA knee samples evaluated with LOOCV (Leave-One Out-Cross Validation). These 12 methylation sites can not only serve as biomarker, but also provide underlying mechanism of OA.

## Introduction

Osteoarthritis is one of the most prevalent chronic joint diseases, characterized by the loss, degeneration and calcification of articular cartilage (Im and Choi, 2013). It often occurs in middle-aged and elderly people, and the incidence in females is significantly higher than that in males (Rushton et al., 2014). Research shows that in recent years, with the quickening pace of life, the number of young people suffering from the disease began to increase, ultimately resulting in sustained growth in the social burden (den Hollander and Meulenbelt, 2015).

Despite the unremitting efforts of many scholars, the pathogenesis of osteoarthritis is still not clear yet. Current studies indicate that osteoarthritis is a common degenerative disease caused by the combination and interaction of many factors such as natural and environmental factors (Felson, 2004). The increase in age, damage of tissue and cell, obesity, the overuse of joints and genetic susceptibility are known as OA major risk factors (Hunter et al., 2002).

Osteoarthritis mainly involves the cartilage of the weight-bearing joints, followed by the synovium. At present, it is generally believed that the central part of OA mechanism is the degeneration of articular cartilage, resulting from the imbalance between anabolism and catabolism in the cartilage extracellular matrix caused by mechanical and biological factors (Martel-Pelletier et al., 2008). Since these changes are reported to be caused by genetic changes in the chondrocyte associated with the OA epigenetic mechanism, it’s assumed that the epigenetic changes of chondrocytes may be a key driver in osteoarthritis (Reynard and Loughlin, 2012).

Studies on epigenetic of OA have suggested that these mechanisms is very significant during the onset and progression of disease (Barter and Young, 2013). Despite the fact that there exist many epigenetic mechanisms like DNA methylation, miRNA and histone modifications (Aref-Eshghi et al., 2015), the involvement of DNA methylation in OA pathophysiology is the most studied subject (Rice et al., 2020; Sun et al., 2020; Zhou et al., 2020). DNA methylation mainly occurs at CpG dinucleotides, selectively adding a methyl group to cytosine to form 5-methycytosine under the catalysis of DNA methyltransferase (DNMT). DNA methylation is involved with transcriptional inhibition by preventing the binding of proteins to gene promoters and changing chromatin structure (Loeser, 2008). The changes of methylation status can accelerate the development of OA (Miranda-Duarte, 2018). Thus, further studies on the mechanisms involved in DNA methylation is another way to develop new OA therapy strategies.

There have been several researches on DNA methylation at specific genes in OA cartilage. For instance, the expression of matrix metalloproteinase genes has been reported to be upregulated in OA chondrocytes, resulting in extracellular matrix degradation (Kevorkian et al., 2004). In addition, genes associated with OA chondrocytes like GDF-5, SOX-9, DIO-2, and ADAMTS-4 were also suggested to be differentially expressed between OA cartilage and control group (Reynard and Loughlin, 2012). It has also been reported that the IL1B promoter is demethylated in articular chondrocytes as a response to inflammatory cytokine signaling (Hashimoto et al., 2009).

OA happens in knees and hips mostly. But knee OA is more common than hip OA. Although articular cartilage of hip and knee joints have substantial similar characters and functions, the disease progression and subsequent treatment may be different between the two joints (Pereira et al., 2016). Transcriptomic studies have indicated that genes dysregulated in hip and knee OA have great difference (Loughlin and Reynard, 2015), so does the methylation patterns in hip and knee OA samples (Rushton et al., 2014). These findings highlight the importance of the separation of OA researches from skeletal sites and help us understand the cartilage homeostasis.

The cure for osteoarthritis is mainly to mitigate pain, improve the function of the joints and avoid the side effects of the treatment as far as possible. However, due to the slow progress of osteoarthritis and the lack of sensitive detection methods to identify early OA changes, it is difficult to find disease-modifying drugs at present. Epigenetic markers mentioned above can detect the phenotypes of various chondrocytes, including articular cartilage homeostasis, chondrogenic differentiation and the development of OA, which may provide new targets and strategies for drug treatment of OA.

To systematically investigate the methylation pattern in knee and hip osteoarthritis, we analyzed the methylation profiles in cartilage of 16 OA hip samples, 19 control hip samples and 62 OA knee samples. 12 discriminative methylation sites were identified based on minimal Redundancy Maximal Relevance (mRMR) and Incremental Feature Selection (IFS). mRMR is a widely used power feature selection method (Chen et al., 2017b, 2018b, 2019c; Cai et al., 2018; Li and Huang, 2018; Li et al., 2019a) which considers not only the relevance with OA status but also the redundancy among methylation status. It can identify a small but well performed methylation signature. What’s more, an SVM (Support Vector Machine) (Chen et al., 2017c, 2019a,b,d; Sun et al., 2018; Li et al., 2019b; Pan et al., 2019) OA classifier was built based on these 12 methylation sites and it can perfectly classify the OA hip samples, control hip samples and OA knee samples evaluated with LOOCV (Leave-One Out-Cross Validation) (Chen et al., 2014; Zhang N. et al., 2015; Cheng et al., 2016; Li et al., 2018; Wang and Huang, 2019). Although the model needs to be validated on independent large dataset, these 12 methylation sites provided clues for the mechanisms of OA.

## Materials and Methods

### The Cartilage DNA Methylation Profiles of OA Hips, Control Hips and OA Knees

We downloaded the cartilage DNA methylation profiles of 16 OA hip samples, 19 control hip samples and 62 OA knee samples from publicly available GEO (Transcript Expression Omnibus) database under accession number of GSE63695 (Rushton et al., 2014). The DNA methylations were measured using Illumina Human Methylation 450 Array. There were 4,82,421 probes corresponding to methylation sites. The processed beta values that were normalized with preprocess Funnorm function from R package minfi were used for further analysis.

### Identify the Representative OA Methylation Sites

To identify the most discriminative features among different groups, many analysis methods have been developed (Huang et al., 2008; Cai et al., 2010; Zhang et al., 2012, 2016, 2017; Li et al., 2014; Zhang P.W. et al., 2015; Chen et al., 2018a; Wang et al., 2018). ANOVA (Analysis of Variance) is an obvious choice. But such statistical methods don’t consider the relationship between features, therefore a lot of redundant features will be selected.

In our study, the number of features, i.e., methylation sites, was extremely large. Obviously, many of them were redundant. To select the representative features, we adopted mRMR method developed by Peng et al. (2005) to reduce redundancy of selected genes. This method has been widely used and proven to be very effective in handling high dimensional data (Niu et al., 2013; Zhao et al., 2013; Zhou et al., 2015; Zhang et al., 2016; Li and Huang, 2017; Liu et al., 2017). The C++ version mRMR program downloaded from http://home.penglab.com/proj/mRMR/was used in this study.

Its idea is simple and clear. Let us use Ω to denote all the 4,82,421 methylation sites, Ω_*s*_ to denote the selected m sites, and Ω_*g*_ to denote the n sites to be selected.

First, we evaluated the relevance of site *g* from Ω_*g*_ with three-class sample labels *l* (OA hips, control hips and OA knees) was calculated with mutual information (*I*) equation (Sun et al., 2012; Huang and Cai, 2013):

(1) I(g,l)

Meanwhile, the redundancy of site *g* with the m selected sites *g*_*i*_ (*i* = 1, 2,…, m) in Ω_*s*_ can also be calculated based on mutual information:

(2) $$\frac{1}{m}\left(\sum_{g_{i}\in {\text{Ω}}_{s}}I\left(g,g_{i}\right)\right)$$

The goal can be characterized as maximizing the function which balances the relevance and redundancy:

(3) $$\max_{g_{j}\in {\text{Ω}}_{g}}\left[I\left(g_{j},l\right)-\frac{1}{m}\left(\sum_{g_{i}\in {\text{Ω}}_{s}}I\left(g_{j},g_{i}\right)\right)\right]\left(j=1,2,\ldots ,n\right)$$

Each time, one best site that maximized this function will be moved from Ω_*g*_ to Ω_*s*_. Eventually, all the sites will be ranked base on their relevance with sample labels and redundancy between each other. The ranked methylation sites from large to small can be represented as:

(4) $$\text{S}=\left\{g_{1}^{\prime },g_{2}^{\prime },\ldots ,g_{r}^{\prime },\ldots ,g_{N}^{\prime }\right\}$$

The top ranked methylation sites have better discriminative ability.

To reduce the computational complexity, we focused on the top 300 mRMR sites for further analysis which should be enough to classify the samples and suitable as biomarkers.

### Optimize the Discriminative Methylation Sites for OA

Although we identified the non-redundant OA sites using mRMR method, we still wanted to obtain the methylation site combinations which can classify the OA hips, control hips and OA knees. To do so, we applied a widely used optimization method, IFS (Jiang et al., 2013; Li et al., 2014; Shu et al., 2014; Zhang et al., 2014; Huang et al., 2015; Zhang P.W. et al., 2015; Chen et al., 2017a).

Based on the ranked mRMR site list, each time, the top r sites $\left\{g_{1}^{\prime },g_{2}^{\prime },\ldots ,g_{r}^{\prime }\right\}$ were chosen to construct a SVM (Support Vector Machine) classifier and its accuracy evaluated with LOOCV (Chen et al., 2014; Zhang N. et al., 2015; Cheng et al., 2016; Li et al., 2018; Wang and Huang, 2019) was recorded. SVM is classical machine learning classifier with a wide range of applications in biomedicine (Chen et al., 2017c, 2019b; Li et al., 2018, 2019b; Sun et al., 2018; Pan et al., 2019). The R function svm in package e1071^1^ was used to apply the SVM method. LOOCV, as known as Jackknife test, is a widely used objective method to evaluate the prediction performance of classifiers (Chou, 2011). Each time, one sample was treated as test sample while the other samples were used to train the model. At last, each sample had been tested for once and the classes of all samples were predicted. By comparing the predicted classes and actual classes, we can calculate the LOOCV accuracy.

After 300 rounds, the performances of the 300 methylation subsets were tested. By analyzing the number of used methylation sites and the performance of corresponding classifier, we can easily find the best methylation sites.

## Results and Discussion

### The Representative OA Methylation Sites

In the OA methylation dataset, there were 4,82,421 features. On one hand, the number of features was large; on the other hand, there were redundancy among these methylation sites. To reduce the number of features to a reasonable number for further study, we adopted the mRMR method.

The methylation sites were ranked based on both their relevance with the sample labels, i.e., OA hips, control hips or OA knees, and their redundancy with each other. In other words, only the methylation site that exhibited different pattern with already selected sites will be selected from the candidate methylation site pool. With the mRMR analysis, we identified the top 300 most representative OA methylation sites.

### The Discriminative Methylation Sites for OA

Although the mRMR method considered the relevance between features and sample labels, but it was balanced with redundancy. We would like to find the methylation sites that can best classify different OA samples. These sites not only should be concise, but also have great discriminative ability.

**FIGURE 1:**
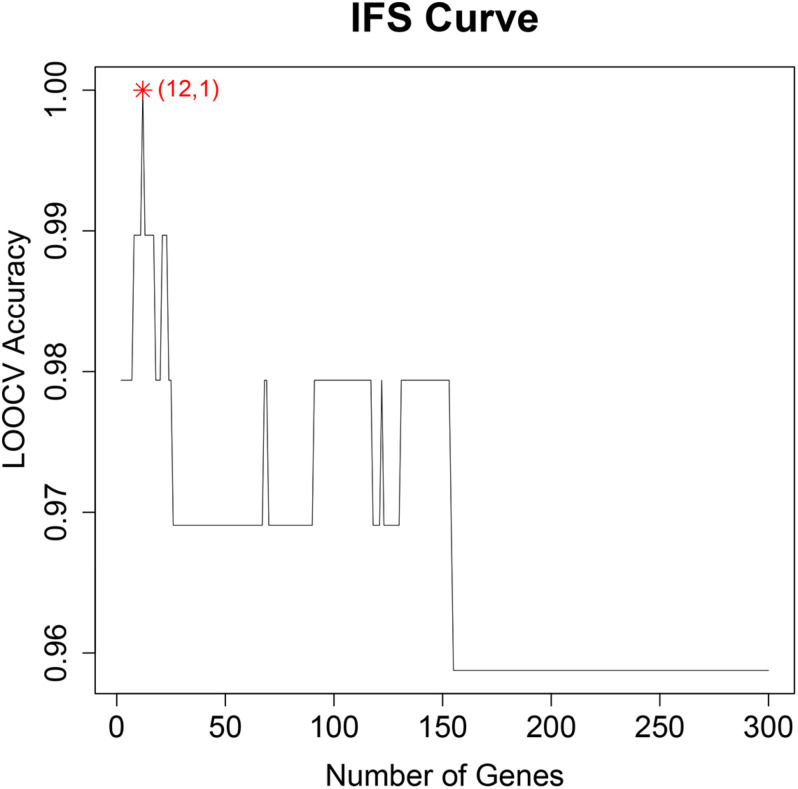
The IFS curve of the number of methylation sites and their classification accuracy. The x-axis was the number of methylation sites used to construct the SVM classifier and y-axis was the classification accuracy of the SVM classifier evaluated with (Leave-One Out-Cross Validation). When the top 12 methylation sites were used, all samples can be perfectly classified.

To find the best discriminative methylation sites for OA, we plotted the IFS results in Figure 1 in which, the *x*-axis was the number of methylation sites and the *y*-axis was the LOOCV accuracy of the SVM classifier based these sites. It can be seen that when the top 12 mRMR sites was used, the ACC was the highest. All samples were correctly classified. The 12 chosen methylation sites were given in Table 1. The chromosome positions were from Genome Build 37 (hg19). We must be cautious that this accuracy needs to be validated on an independent large OA cohort. But since there is no other similar dataset, we can only try our best and evaluate it with objective LOOCV method. The results provided clues about the difference between OA and control and the difference between hip OA and knee OA and worth to be further investigated.

**TABLE 1:** The 12 discriminative methylation sites for OA.

Rank	Probe	Chromosome	Coordinate	Gene	mRMR score
1	cg09462924	2	66666470	MEIS1	0.943
2	cg22118147	5	172144013	-	0.263
3	cg18576667	15	27597409	GABRG3	0.328
4	cg07533951	12	114879558	-	0.284
5	cg14545975	9	137297213	RXRA	0.245
6	cg05877497	2	66667946	MEIS1	0.264
7	cg21811143	2	119599748	EN1	0.219
8	cg09989996	1	753376	FAM87B	0.235
9	cg19738283	2	176976802	HOXD10	0.244
10	cg02824888	12	115129011	-	0.226
11	cg04288999	2	66667852	MEIS1	0.238
12	cg00995986	2	66665428	MEIS1	0.214

**FIGURE 2:**
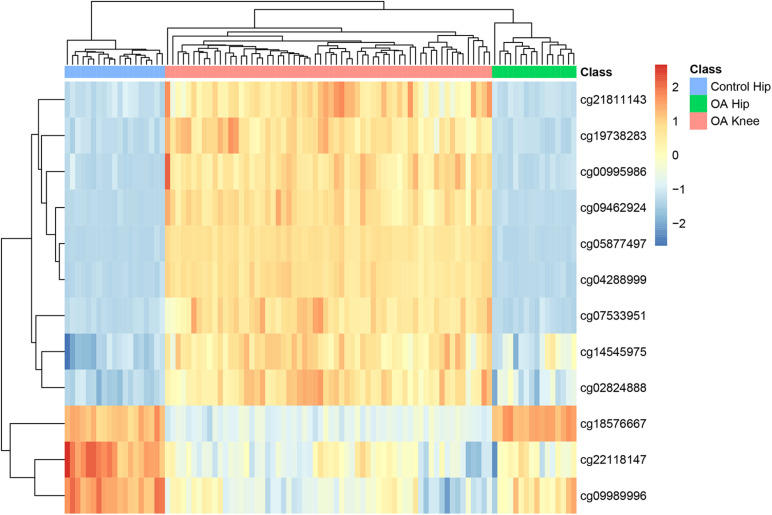
The heatmap of the 12 methylation sites in OA hips, control hips and OA knees. Each row corresponded to the scaled methylation level of one site and each common corresponded to a sample which could be OA hip, control hip or OA knee. It can be seen that all samples were clustered into the right groups.

To explore the methylation levels of the 12 sites in different disease status, we plotted the heatmap of them in all samples in Figure 2. It can be seen that OA hip were more similar with OA knee than with control hip. The disease status surpassed the tissue specificity. As for OA hip and OA knee, there were nine highly methylated sites in OA knee (cg21811143, cg19738283, cg00995986, cg09462924, cg05877497, cg04288999, cg07533951, cg14545975, and cg02824888) while there were three highly methylated sites in OA hip (cg18576667, cg22118147, and cg09989996).

### The Relationship Between the Methylation Signature of Cartilage Tissue and Gene Expression Signature of Blood in OA

**FIGURE 3:**
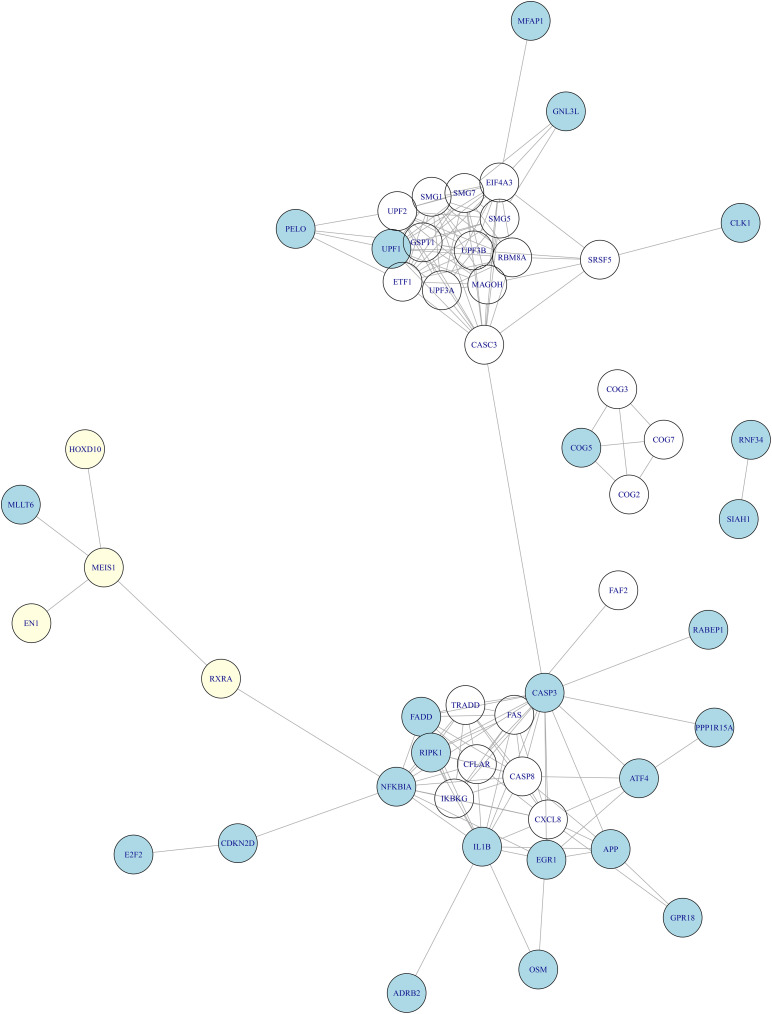
The relationship between the methylation signature of cartilage tissue and gene expression signature of blood in OA. We mapped the tissue methylation signature genes (light yellow) and blood expression signature genes (light blue) onto the STRING network and found that MEIS1 was the hub of methylation signature which can interact with the other three methylation genes (EN1, RXRA, and HOXD10) and the blood expression signature gene (MLLT6). Through RXRA, MEIS1 can regulate a large cluster of blood expression signature genes.

There have been studies of blood expression profiles of OA (Ramos et al., 2014; Li et al., 2018). Ramos et al. identified 27 blood expression signature genes of OA (Ramos et al., 2014) and Li et al. (2018) identified 23 blood expression signature genes. By combining them, we obtain 46 blood expression signature genes. There was no overlap between the 46 blood expression signature genes and our cartilage tissue methylation signature genes. But when we mapped them onto STRING network^2^ (Szklarczyk et al., 2018), the network relationship between tissue methylation genes and blood expression genes (Figure 3) was clear. It can be seen that MEIS1 was the hub of methylation signature which can interact with the other three methylation genes (EN1, RXRA, and HOXD10) and the blood expression signature gene (MLLT6). Through RXRA and MEIS1 can regulate a large cluster of blood expression signature genes.

### The Biological Functions of the Discriminative Methylation Sites for OA

**FIGURE 4:**
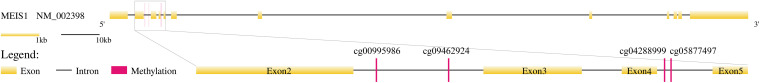
The four methylation sites on the gene structure of MEIS1. MEISI is a long gene of 1,40,418 bps with 13 exons. The methylation sites cg00995986 and cg09462924 located between exon 2 and exon 3 while cg04288999 and cg05877497 located between exon 4 and exon 5 in MEIS1. They were close on chromosome position.

In Table 1, we annotated the methylation sites to genes based on their chromosome coordinates. Four of them were corresponding to the same gene, MEIS1 (Meis Homeobox 1). We checked the consistency of these four MEIS1 methylation sites in OA hips, control hips and OA knees in Figure 2 and we found they shared similar pattern although they were not identical. MEISI is a long gene of 1,40,418 bps with 13 exons. We mapped the four methylation sites onto the gene structure of MEIS1 (Figure 4). The methylation sites cg00995986 and cg09462924 located between exon 2 and exon 3 while cg04288999 and cg05877497 located between exon 4 and exon 5. They were close on chromosome position. There have been reports that MEISI was associated with blood and epithelial cancers, such as acute leukemia and skin cancer (Okumura et al., 2014; Wang et al., 2014). As a transcription factor, MEISI can bind chromatin DNA and regulate pluripotency of stem cells (Okumura et al., 2014). It is generally considered as an oncogene which accelerate development (Wang et al., 2014). There are no reported associations between MEISI and osteoarthritis. MEISI may serve as a novel OA biomarker, especially for distinguishing the difference between hip OA and knee OA.

Another interesting methylation site was cg21811143 which locate in EN1 (Engrailed Homeobox 1). It plays a role in controlling development of the central nervous system (CNS) (Webb et al., 2008) and segmentation course, where it is required for the formation of posterior compartments (Lawrence and Johnston, 1984). Recent studies have identified EN1 gene as a decisive factor of bone mineral density (BMD) via whole-genome sequencing (Zheng et al., 2015), while higher BMD was considered to be involved with OA in many cross-sectional and longitudinal epidemiological studies (Belmonte-Serrano et al., 1993). SNP rs4144782 in EN1 was reported to be significantly associated with increased risk of knee OA (OR = 1.26; 95% CI: 1.05–1.50, *p*-value = 0.012) (Li et al., 2017). What’s more, the EN1 mRNA levels were differentially expressed between Knee and hip Intra-articular adipose tissues (IAATs) and Subcutaneous adipose tissue (SCAT) (Eymard et al., 2017). Its expression was strongly decreased in all IAATs compared with SCAT (Infrapatellar fat pad, IFP: 0.3-fold, *p* = 0.006; Suprapatellar fat pad, SPFP: 0.2-fold, *p* = 0.006; and Acetabular fat pad, AFP: 0.3-fold, *p* = 0.046) (Eymard et al., 2017). The genomic, methylation and transcriptomic evidences of the association between EN1 and OA make it a strong candidate OA gene worth further experimental validation.

GABRG3 and RXRA, were gamma-aminobutyric acid (GABA) receptor and retinoid X receptor, respectively. Beside autistic disorder (Menold et al., 2001) and Alzheimer’s disease (Iwakiri et al., 2009), GABRG3 is associated with alcohol dependence by affecting disinhibition and hyperexcitability of CNS (Dick et al., 2004; Edenberg and Foroud, 2006). Maybe it can also affect how OA patients feel about pain. For RXRA, its methylation status was reported to be associated with childhood bone mineral content (BMC) (Harvey et al., 2014).

The methylation sites that can’t be annotated to genes may functions through trans-regulation (van Eijk et al., 2012). Their roles can be investigated with integrative analysis of multi-omics OA data in the future.

Another issue of this work was the small sample size. It will overestimate the prediction performance. The confounding factors and disease heterogeneity will be difficult to detect in such small dataset. The signature needs to be validated on large cohorts from different medical centers.

## Conclusion

As a chronic joint disease, osteoarthritis is very common in elder people. Even young people are more and more likely to have OA symptoms due to the quickening pace of life. OA happens in knees and hips mostly, but knee OA is more common than hip OA. The disease progression and treatment of hip and knee OA are different. Since the epigenetic factor plays key roles in OA, we systematically analyzed the DNA methylation profiles of cartilage from 16 OA hip samples, 19 control hip samples and 62 OA knee samples. With advanced feature selection methods, 12 OA discriminative methylation sites were selected from a total of 4,82,421 sites. These sites corresponded to genes like MEIS1, EN1, GABRG3, and RXRA. These results provided not only novel OA biomarkers, but also possible mechanisms that worth further investigation in an independent large cohort.

## Data Availability Statement

Publicly available datasets were analyzed in this study. This data can be found here: https://www.ncbi.nlm.nih.gov/geo/query/acc.cgi?acc=GSE63695.

## Author Contributions

TH and XX designed the experiment. ZW performed the experiment. LS and JW participant in the data analysis. ZW, TH, and XX wrote the manuscript. All authors contributed to the article and approved the submitted version.

## Conflict of Interest

The authors declare that the research was conducted in the absence of any commercial or financial relationships that could be construed as a potential conflict of interest.

## Footnotes

1 https://CRAN.R-project.org/package=e1071

2 http://string-db.org, version: 11.0

## Abbreviations

mRMR: minimal Redundancy Maximal Relevance
SVM: Support Vector Machine
IFS: Incremental Feature Selection
DNMT: DNA methyltransferase
LOOCV: Leave-One Out-Cross Validation
GEO: Transcript Expression Omnibus.
